# Application of Population Pharmacokinetic Analysis to Characterize CYP2C19 Mediated Metabolic Mechanism of Voriconazole and Support Dose Optimization

**DOI:** 10.3389/fphar.2021.730826

**Published:** 2022-01-03

**Authors:** SiChan Li, SanLan Wu, WeiJing Gong, Peng Cao, Xin Chen, Wanyu Liu, Liping Xiang, Yang Wang, JianGeng Huang

**Affiliations:** ^1^ Department of Clinical Pharmacy, Wuhan Children’s Hospital (Wuhan Maternal and Child Healthcare Hospital), Tongji Medical College, Huazhong University of Science and Technology, Wuhan, China; ^2^ Department of Pharmacy, Union Hospital, Tongji Medical College, Huazhong University of Science and Technology, Wuhan, China; ^3^ Hubei Province Clinical Research Center for Precision Medicine for Critical Illness, Wuhan, China; ^4^ School of Pharmacy, Tongji Medical College, Huazhong University of Science and Technology, Wuhan, China

**Keywords:** voriconazole, voriconazole N-oxide, population pharmacokinetics, CYP2C19, genetic polymorphism

## Abstract

**Purpose:** The aims of this study were to establish a joint population pharmacokinetic model for voriconazole and its N-oxide metabolite in immunocompromised patients, to determine the extent to which the *CYP2C19* genetic polymorphisms influenced the pharmacokinetic parameters, and to evaluate and optimize the dosing regimens using a simulating approach.

**Methods:** A population pharmacokinetic analysis was conducted using the Phoenix NLME software based on 427 plasma concentrations from 78 patients receiving multiple oral doses of voriconazole (200 mg twice daily). The final model was assessed by goodness of fit plots, non-parametric bootstrap method, and visual predictive check. Monte Carlo simulations were carried out to evaluate and optimize the dosing regimens.

**Results:** A one-compartment model with first-order absorption and mixed linear and concentration-dependent-nonlinear elimination fitted well to concentration-time profile of voriconazole, while one-compartment model with first-order elimination well described the disposition of voriconazole N-oxide. Covariate analysis indicated that voriconazole pharmacokinetics was substantially influenced by the *CYP2C19* genetic variations. Simulations showed that the recommended maintenance dose regimen would lead to subtherapeutic levels in patients with different CYP2C19 genotypes, and elevated daily doses of voriconazole might be required to attain the therapeutic range.

**Conclusions:** The joint population pharmacokinetic model successfully characterized the pharmacokinetics of voriconazole and its N-oxide metabolite in immunocompromised patients. The proposed maintenance dose regimens could provide a rationale for dosage individualization to improve clinical outcomes and minimize drug-related toxicities.

## Introduction

Voriconazole (VCZ) is a second-generation triazole with an expanded spectrum of activity against invasive fungal, including Aspergillus, Scedosporium, *Fusarium*, and resistant Candida species (Ghannoum and Kuhn, 2002). It has been approved for systemic prophylaxis and treatment of a variety of invasive fungal infections (IFIs) in adults and children. IFIs usually occur in hospitalized or immunocompromised patients and could lead to substantial morbidity and mortality (Sung et al., 2009; Fisher et al., 2018). Patients who suffered from different forms of hematological disorders or received immunosuppressive therapy are highly susceptible to IFIs caused by molds and yeasts (Lehrnbecher et al., 2020).

Following oral administration, VCZ is rapidly and almost completely absorbed, with a high bioavailability of 96% (Schulz et al., 2019). Hence, switching between intravenous and oral administration without dose adaptation is permitted in clinical practice. It is extensively distributed throughout the body and exhibits a concentration- and dose-independent plasma protein binding of 58%. VCZ undergoes N-oxidative metabolism in the liver, predominantly by the CYP2C19 isoenzyme and to a lesser extent by CYP2C9 and CYP3A4. Only less than 2% of the dose is excreted via urine as unchanged drug (Theuretzbacher et al., 2006). Voriconazole N-oxide (VNO) is the major metabolite in the circulation and accounts for more than 70% of the circulating metabolites in the plasma. As indicated in previous *in vitro* studies, although VNO showed little anti-fungal activity, it was found to have inhibitory effects on CYP2C19 and CYP3A4 activities (Jeu et al., 2003). Simultaneous determination of VNO and VCZ may be useful in elucidating the primary metabolic process of VCZ and evaluating the potential side effects of VNO.

VCZ exhibits highly variable inter- and intra-individual pharmacokinetics, which may limit its potential application in clinical conditions (Dolton et al., 2014). Numerous factors could be responsible for this variability, including body weight, age, gender, altered gastrointestinal absorption, pharmacogenetic polymorphisms, drug interactions, chemotherapy and inflammation status (Stott and Hope, 2017; Mafuru et al., 2019; Vena et al., 2020). Besides, VCZ follows classical nonlinear pharmacokinetics as a result of saturation of metabolic clearance and dose-dependent auto-inhibition (Yamada et al., 2015; Schulz et al., 2019).Considering the high risk of mortality associated with IFIs and large inter-individual variations in VCZ pharmacokinetics, therapeutic drug monitoring (TDM) is recommended as a promising strategy to optimize anti-fungal therapy (Yi et al., 2017).The relationship between VCZ exposure and clinical response has been established in previous studies. A VCZ plasma trough level of 1.0–5.5 mg/L (Pascual et al., 2012; Ashbee et al., 2014) or 2–5 mg/L (Dolton et al., 2012) is generally accepted as the target range for improved clinical outcomes and minimized adverse effects (hepatotoxicity, visual disorder, neurotoxicity) (Jin et al., 2016).

*CYP2C19* polymorphisms could explain a substantial part of the remarkable inter-individual variability in VCZ pharmacokinetics (Weiss et al., 2009). The major variants of the *CYP2C19* alleles in the Chinese population are *1, *2, *3, and *17 (Moriyama et al., 2017). The wild type (CYP2C19*1) is the most frequently found in our population, with a prevalence of about 60% (Fu et al., 2004; Wang et al., 2007; Zuo et al., 2012). The genotypic distributions of *CYP2C19* in Chinese leads to phenotypes of poor metabolizers (PM), intermediate metabolizers (IM), normal metabolizers (NM),rapid metabolizers (RM)and ultrarapid metabolizers (UM). The CYP2C19*2 and *3 alleles are loss-of-function alleles and play an important role in intermediate metabolism in many Chinese patients. The frequencies of IMs and PMs were 45.62 and 13.42% in Chinese, respectively, which are much higher than those in Caucasian, African or American population. Moreover, the increased function CYP2C19*17 allele is rare in Chinese people (4%) (Sim et al., 2006). Although rapid metabolism will result in low VCZ levels, the frequency of RMs in Chinese (1.06%) is relatively low when compared to other ethnic populations (He et al., 2020). Clinical studies demonstrated that PMs will achieve 2–4 times higher VCZ exposure than NMs, and exhibit a higher risk of drug-related toxicities (Dean, 2012; Chawla et al., 2015).Therefore, dose alteration may be required in some cases, and TDM combined with pharmacogenetic testing would be an effective approach for individualized dose adjustment of VCZ.

As far as we know, relatively few studies have simultaneously described the pharmacokinetics of VCZ and its N-oxide metabolite in immunocompromised patients. Therefore, this study intended to develop a joint population pharmacokinetic (PopPK) model of VCZ and VNO, and to determine the contribution of genetic polymorphisms in *CYP2C19* to the variability in VCZ pharmacokinetics, which facilitated individualized therapies based on genotype.

## Methods

### Study Design and Patient Population

This single-centre, retrospective pharmacokinetic study of VCZ was conducted at Union Hospital in Wuhan, China, from Feb 2017 to July 2018. The inclusion criteria were as follows: 1) age>12 years old; 2) patients treating with oral VCZ for IFIs (possible, probable or proven). The exclusion criteria included: 1) intolerant to VCZ treatment; 2) critical data were missing; 3) participated in another clinical trial simultaneously.

This study was approved by the Ethics Committee of Tongji Medical College, Huazhong University of Science and Technology (No: IORG0003571) and written informed consent was obtained from all patients.

### Drug Administration, Blood Sampling and Data Collection

VCZ was administered orally at a dose of 200 mg twice daily without a loading dose. Whole blood samples (5 ml) were collected for routine therapeutic drug monitoring of VCZ trough concentration and *CYP2C19* genotyping. Most samples were taken when steady state was attained after 5 days of dosing, while others were collected and tested when the physicians deemed it necessary. The samples were drawn into EDTA-K_2_-containing tubes before the following dose administration. The plasma was obtained after 15 min centrifugation under 1500 g and stored at –80°C until assay.

Demographic and physiological information of all patients were extracted from the electronic medical records database, including gender, age, height, body weight (WT), total bilirubin (TBIL), direct bilirubin (DBIL), indirect bilirubin (IBIL), total bile acids (TBA), alanine aminotransferase (ALT), aspartate aminotransferase (AST), alkaline phosphatase (ALP), gamma-glutamyl transferase (GGT), total protein (TP), albumin (ALB), globulin (GLB), blood urea nitrogen (BUN), uric acid (UA), serum creatinine concentration (SCR). The body surface area (BSA) was calculated based on the Mosteller formula. In addition, concomitant medications (proton-pump inhibitors and glucocorticoids) were recorded.

### Quantification of VCZ and VNO in Plasma

A liquid-liquid extraction (LLE) method using methyl tert-butyl ether (MTBE) as the extraction solvent was applied for sample preparation. Patient plasma (95 μL) was spiked with 5 μL of 80% MeOH followed by the addition of 10 μL internal standard (IS, propranolol). Then, 1,000 μL MTBE was added into the above mixture and vortexed for 3 min at 4°C. After centrifugation at 12000 *g* for 10 min, 800 μL supernatant was aspirated and dried in a clean tube under vacuum condition and then reconstituted in 80 μL of acetonitrile. Finally, 10 μL of the processed sample was injected into the liquid chromatography-tandem mass spectroscopy (LC-MS/MS) system for analysis.

Liquid chromatography was performed in a Shimadzu Prominence UFLC system (Shimadzu Corporation, Kyoto, Japan) with an Ultimate UHPLC XB-C18 column (50 mm × 2.1 mm, 1.8 μm, Welch, China) at 35°C. The mobile phase, pumped at a flow rate of 0.35 ml/min, consisted of 2 mM ammonium formate (mobile phase A) and acetonitrile (mobile phase B). The gradient elution conditions were as follows: 0–0.1 min, 10% B; 0.1–0.5 min, 10–50% B; 0.5–1.0 min, 50% B; 1.0–1.1 min, 50–60% B; 1.1–3.0 min, 60% B; 3.0–3.1 min, 60–10% B; 3.1–4.0 min, 10% B. An API-4000 Q Trap triple quadrupole mass spectrometer (AB Sciex, Foster City, CA, United States) was used for detection of the analytes. After optimization, tandem mass spectrometric detections were performed under the following operational parameters: curtain gas, 10 psi; collision-activated dissociation gas, 4; ion spray voltage, 5,500 V; source temperature, 500°C; gas 1, 50 psi; gas 2, 50 psi; interface heater, on. The quantification was accomplished by electrospray ionization in positive ion mode with multiple reaction monitoring (MRM). The MRM transitions were m/z 350.1→280.8, m/z 366.0→142.9 and m/z 260.1→116.0 for VCZ, VNO and IS, respectively. The lower limit of quantification was 0.5 ng/ml for both analytes. The calibration curves were linear over a range of 0.5–100 ng/ml for both analytes. The intra- and inter-day precision determined by coefficients of variation were less than 10% for both analytes. The accuracies for VCZ and VNO were 89.2–109.8% and 88.1–94.3%, respectively.

### Genotype Analysis

Total Genomic DNA was isolated from whole blood samples with QIAamp DNA blood kits (Qiagen, Hilden, Germany) according to the instruction supplied by the manufacturer. Genomic polymorphisms of CYP2C19*2 (c.681G > A, rs 4244285), CYP2C19*3 (c.636G > A, rs4986893), and CYP2C19*17 (c.-806 C > T, rs12248560) were identified by polymerase chain reaction-restriction fragment length polymorphism (PCR-RFLP) method on an ABI 3730XL Genetic Analyzer (Applied Biosystems, Foster City, CA, United States) as previously described (Mafuru et al., 2019). When *CYP2C*19*2 and *3 alleles were not detected, the allele was identified to be CYP2C19 *1 (wild type). All variant alleles were in accordance with Hardy-Weinberg equilibrium. The CYP2C19 phenotype was determined according to the guideline by the Clinical Pharmacogenetics Implementation Consortium (CPIC) (Moriyama et al., 2017). The UM and RM were defined as *17/*17 homozygote and *1/*17 heterozygote, respectively. And the NM was assigned to patients with deficient allele heterozygote (e.g., *1/*1). The IM was defined as a patient carrying one loss-of-function allele in combination with one normal function allele (e.g., *1/*2, *1/*3, *2/*17). Besides, the PM was defined as a patient with deficient allele compound heterozygote or homozygote (e.g.,*2/*2, *2/*3 or *3/*3).

### PopPK Modelling

A PopPK analysis was conducted using the software Phoenix^®^ NLME (Version 8.2.0.4383, Pharsight Corporation, United States) to estimate the pharmacokinetic parameters of VCZ. The first-order conditional estimation-extended least squares (FOCE ELS) algorithm was applied throughout the modeling procedure. The R program (version 4.0.2, http://www.r-project.org/) was utilized for data visualization and model validation.

### Base Model

All VCZ concentrations in the present study were trough concentration data, which could not be used for characterization of the absorption phase. Thus, according to data from published literature, the absorption rate constant (k_a_) and the bioavailability (F) were fixed at 1.1/h and 0.895, respectively (Pascual et al., 2012; Wang et al., 2014). Considering the limited sampling times, one-compartment models based on first-order absorption with either linear or non-linear (Michaelis–Menten) elimination were tested to fit the concentration profile of VCZ. In addition, compartmental and residual error models for VNO were identified to describe the metabolic pathway from VCZ to its N-oxide metabolite.

The inter-individual variability in pharmacokinetic parameters was estimated using an exponential function as follows:

$$P_{i}=\text{θ×exp}\left(\eta_{i}\right)$$ (1)

Where *P*_*i*_ represents the estimated pharmacokinetic parameter for individual *i*, *θ* is the population typical value of that parameter, and η_*i*_ represents the random variable for individual *i*, which is defined as normally distributed with a mean of 0 and a variance of *ω* ^2^.

The residual variability was evaluated by additive, proportional or combined additive-proportional residual error models. The equations were as follows:

$$C_{ij}=C_{pred,ij}+\varepsilon_{ij}$$ (2)

$$C_{ij}=C_{pred,ij}\times \left(1+\varepsilon_{ij}\right)$$ (3)

$$C_{ij}=C_{pred,ij}\times \left(1+\varepsilon_{ij1}\right)+\varepsilon_{ij2}$$ (4)

Where *C*_*ij*_ is the *j*th observed value in individual *I*; *C*_*pred,ij*_ is the *j*th predicted value in individual *i*; *ε*_*ij*_is the residual random error, which is assumed to be Gaussian distributed with a mean of 0 and a variance of *σ* ^2^.The selection of the appropriate structural model was based on the visual inspection of diagnostic plots and improvements of the statistic parameters, including Akaike information criterion (AIC), Bayesian information criterion (BIC) and the objective function value (OFV).

### Covariate Model

After development of the base model, the potential covariates were tested using stepwise forward selection followed by backward elimination steps. Pairwise correlations between all variables were assessed prior to covariate analysis, and highly collinear variables (correlation coefficient >0.5) would not be simultaneously incorporated into the final model. Covariates associated with a significant decrease of OFV more than 3.84 units (Chi-squared distribution, df = 1, *p* ≤ 0.05) were added to the base model to establish a full model. Then the covariates were removed from the full model one by one. Covariates resulting in a significant increase of OFV by at least 6.63 units (Chi-squared distribution, df = 1, *p* ≤ 0.01) were retained in the final model. The possible influences of all demographic and physiological variables were explored. In the screening process, power models (Eq. (5)) were used for continuous covariates such as age, body weight, BSA, renal and liver function parameters. While exponential models (Eq. (6)) were used for categorical covariates such as sex, concomitant medications, andCYP2C19 metabolic phenotypes.

$$\theta_{i}=\theta \times {\left(\frac{Cov_{j}}{Cov_{median}}\right)}^{\theta_{cov}}$$ (5)

$$\theta_{i}=\theta \times \exp\left(\theta_{\mathrm{cov}}\right)$$ (6)

where Cov_j_ is the *j*th covariate, Cov_median_ is the median value of covariate, θ_i_ is the population prediction of the pharmacokinetic parameter, *θ* is the population typical value of the parameter, and θ_cov_describes the fixed effect of the covariate on the parameter.

A post hoc empirical Bayesian method was employed to estimate individual exposure pharmacokinetic parameters at steady state for both VCZ and VNO, including trough concentration (C_min_), peak concentration (C_max_) and the area under drug plasma concentration-time curve over 24 h at day 7 (AUC, 144–168 h) with twice-daily oral administration of 200 mg VCZ. Additionally, metabolic ratio (MR) was calculated using Equation (7) (Yamada et al., 2015). SPSS software version 19.0 (SPSS Inc. Chicago, IL, United States) was used for exploratory data analysis.

$$\text{MR}=\frac{{\text{AUC}}_{\text{VNO}}}{{\text{AUC}}_{\text{VCZ}}}$$ (7)

where AUC_VNO_ and AUC_VCZ_ are the area under drug plasma concentration-time curve of VNO and VCZ, respectively.

### Model Validation

The goodness of fit plots, nonparametric bootstrap method, and visual predictive check (VPC) were employed for model validation. Different diagnostic plots such as observed concentrations (DV) vs individual predictions (IPRED), DV vs population predictions (PRED), Conditional weighted residuals (CWRES) vs time, and CWRES vs PRED graphs were drawn to visually assess the accuracy of the PopPK model. A nonparametric bootstrap procedure was carried out using 1,000 randomly resampled datasets generated from the original data. To evaluate model stability, the 2.5th, 50th, and 97.5th percentile of the bootstrap estimates were computed and compared to the final model parameter estimates. The VPC method was performed for both VCZ and VNO by simulating 1,000 virtual subjects based on the final model estimates. The simulated concentration profiles were graphically compared with observed concentrations to evaluate the predictive performance of the final model.

### Model-Based Simulations

The Monte Carlo simulations were performed based on the parameter estimates derived from the final model. Each simulation runs for 1,000 times to predict the VCZ concentration profiles following multiple oral doses. To evaluate and optimize the currently used dosing regimen, oral maintenance doses of 150–400 mg twice daily (BID) or three times daily (TID) were simulated in patients with various CYP2C19 phenotypes. Different concentration cut-off values for efficacy and toxicity (1, 2 and 5.5 mg/L) were employed to determine the probability of supratherapeutic or subtherapeutic levels with each dosing scenario.

## Results

### Patient Characteristics

**FIGURE 1:**
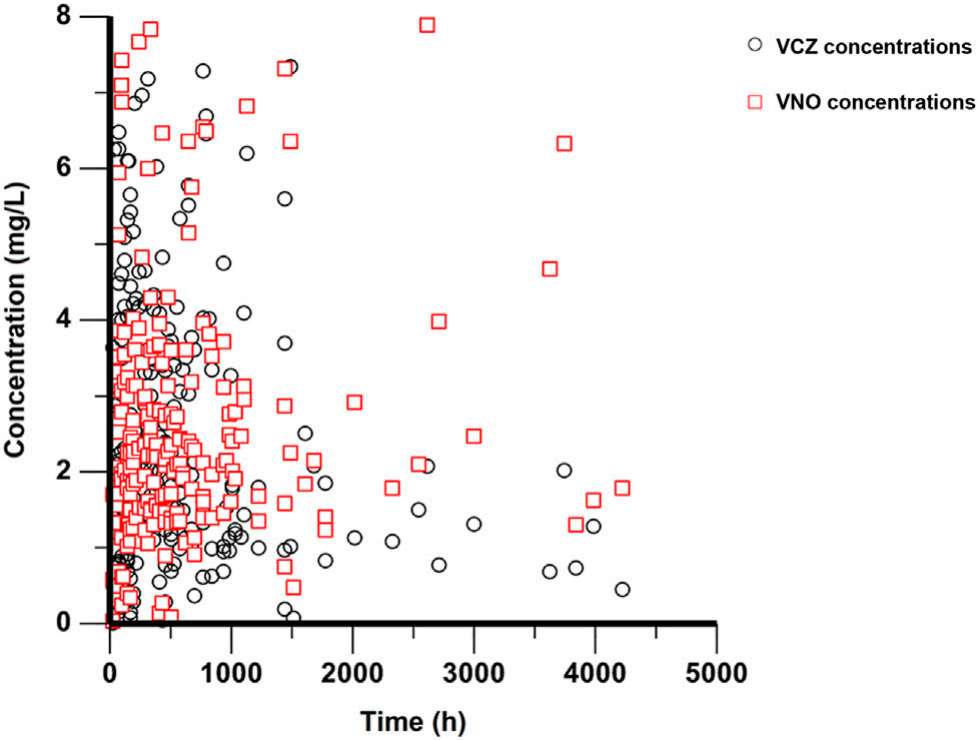
Plasma concentration-time profiles of VCZ and VNO. Black circles represent VCZ concentrations and red squares represent VNO concentrations.

A total of 78 patients participated in this study and no patient was excluded from PopPK analysis. These patients ranged in age from 14 to 70 years and in weight from 44 to 111 kg. Of these patients, only four were adolescents between the ages of 14 and 17, while the rest were adults. All participants provided 214 plasma concentrations for VCZ (range 0.01–7.34 μg/ml) and 213 plasma concentrations for VNO (range 0.04–7.89 μg/ml) measured, with a maximum of eight samples per patient. The sampling time after the first dose ranged from 23 to 4,223 h. The plasma concentration versus time profiles for both VCZ and VNO are shown in Figure 1. The genotyping results were obtained from 75 patients, the majority (n = 32) of them were categorized as IMs, 27 as NMs, and 16 as PMs. The CYP2C19*17 allele was not detected in the genotype analysis, which may be due to the limited frequency of the *17allele in Chinese population (Sim et al., 2006) or the sample size of our study. The remaining three patients were excluded from subgroup comparison analysis due to lack of genotyping information. The demographic and clinical characteristics of all patients are summarized in Table 1.

**TABLE 1:** Demographic and clinical characteristics of the study population.

—	Number	Mean ± SD	Median (range)
Patients	78	—	—
Gender (M**:**F)	57:21	—	—
Age (years)	—	37.5 ± 14.7	36.5 (14.0–70.0)
WT (kg)	—	63.2 ± 12.3	64.0 (44.0–111.0)
Height (cm)	—	167.6 ± 6.0	170.0 (151.0–190.0)
BSA (m^2^)	—	1.709 ± 0.175	1.738 (1.414–2.310)
VCZ concentration (μg/ml)	214	2.47 ± 1.78	2.02 (0.01–7.34)
VNO concentration (μg/ml)	213	2.52 ± 1.59	2.14 (0.04–7.89)
Laboratory parameter			
BUN (mmol/L)	—	7.71 ± 5.90	5.92 (1.36–35.21)
UA (μmol/L)	—	273.2 ± 128.2	267.1 (1.7–677.6)
SCR (μmol/L)	—	84.0 ± 63.7	72.4 (30.4–667.7)
TBIL (μmol/L)	—	11.3 ± 7.8	9.7 (2.9–60.1)
DBIL (μmol/L)	—	6.1 ± 6.2	4.6 (0.9–50.6)
IBIL (μmol/L)	—	5.4 ± 2.9	4.8 (0.8–25.9)
TBA (μmol/L)	—	10.6 ± 11.6	7.0 (1.0–86.1)
ALT (U/L)	—	27.8 ± 33.5	17.0 (3.0–256.0)
AST (U/L)	—	25.7 ± 29.8	18.0 (3.0–261.0)
ALP (U/L)	—	134.4 ± 120.3	100.0 (30.0–898.0)
GGT (U/L)	—	122.4 ± 182.1	65.0 (10.0–1,445.0)
TP (g/L)	—	60.6 ± 9.5	61.5 (30.1–84.7)
ALB (g/L)	—	39.1 ± 6.4	39.5 (20.0–51.8)
GLB (g/L)	—	21.6 ± 5.1	21.2 (7.9–39.1)
Comedication, (%)			
Proton-pump inhibitor^a^	—	51 (100)	46.7%
Glucocorticoids^a^	—	33 (70)	32.7%

WT, body weight; BSA, body surface area; VCZ, voriconazole; VNO, voriconazole N-oxide; BUN, blood urea nitrogen; UA, uric acid; SCR, serum creatinine concentration; TBIL, total bilirubin concentration; DBIL, direct bilirubin; IBIL, indirect bilirubin; TBA, total bile acids; ALT, alanine aminotransferase concentration; AST, aspartate aminotransferase concentration; ALP, alkaline phosphatase; GGT, gamma-glutamyl transferase; TP, total protein; ALB, albumin; GLB, globulin.

a Presented as number of patients (samples) and percentage of samples.

### PopPK Model Development

**FIGURE 2:**
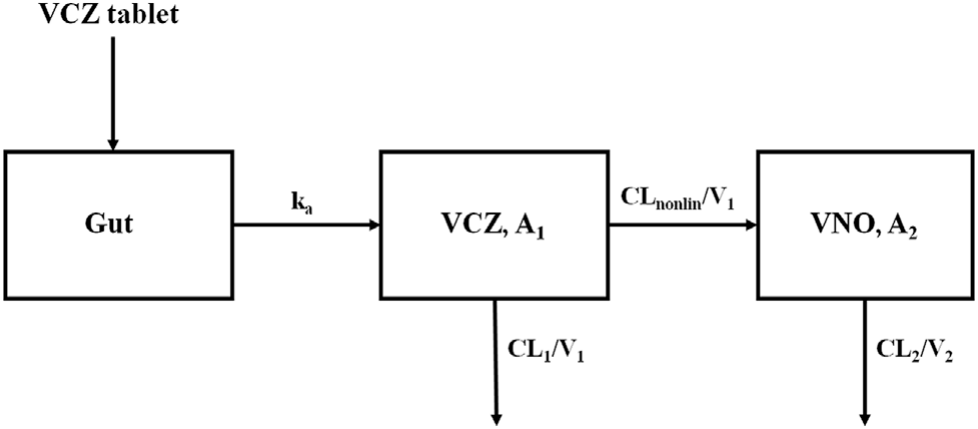
Scheme of the structural model used to describe plasma concentration-time profiles of VCZ and VNO.

The concentration-time profile of VCZ was well fitted to a one-compartment model with first-order absorption and mixed linear and concentration-dependent nonlinear (Michaelis-Menten) elimination. Meanwhile, the disposition of VNO was adequately described by a one-compartment model, with an input rate the same as the conversion rate from VCZ to VNO (Figure 2). For both VCZ and VNO, the inter-individual variability and the residual variability could be best expressed using an exponential model and a proportional model, respectively. In this joint pharmacokinetic model, VCZ was represented by a central compartment (A_1_), parameterized by the nonlinear clearance of VCZ to the metabolite VNO (CL_nonlin_), the clearance of VCZ other than the metabolic pathway converting to VNO (CL_1_), and the volume of distribution (V_1_). VNO was described by a single metabolite compartment (A_2_), in which the clearance (CL_2_) and the distribution volume (V_2_) of VNO were used. Hence, the equations that illustrated the final structural model were as follows:

$$\frac{dA_{gut}}{dt}=-k_{a}\times A_{gut}$$ (8)

$$\frac{dA_{1}}{dt}=F\times A_{gut}\times k_{a}-\frac{CL_{1}}{V_{1}}\times A_{1}-\frac{CL_{nonlin}}{V_{1}}\times A_{1}$$ (9)

$$\frac{dA_{2}}{dt}=\frac{CL_{nonlin}}{V_{1}}\times A_{1}\times k_{n}-\frac{CL_{2}}{V_{2}}\times A_{2}$$ (10)

$$CL_{nonline}=\frac{V_{\max}}{C_{1}+k_{m}}\times \left(1-I_{max}\cdot \frac{C_{2}}{IC_{50}+C_{2}}\right)$$ (11)

$$C_{1}=\frac{A_{1}}{V_{1}}$$ (12)

$$C_{2}=\frac{A_{2}}{V_{2}}$$ (13)

Where A_gut_ is the amount of VCZ in the absorption site, k_a_ is the absorption rate constant, F is the oral bioavailability, V_max_ is the maximum elimination rate, K_m_ is the Michaelis-Menten constant, C_1_ and C_2_ represent the plasma concentrations of VCZ and VNO, respectively. I_max_ is the maximal inhibitory effect, IC_50_ is the concentration of VNO yielding 50% of maximum clearance inhibition. In this model, k_m_, I_max_, and IC_50_ were fixed at 1.15 mg/L, 0.75 and 14.6 mg/L, respectively (Liu et al., 2014; Hohmann et al., 2017).

For basic model, the OFV, AIC and BIC were 1,320.48, 1,342.48 and 1,387.11, respectively. Covariate screening analysis indicated that the age, gender, WT and BSA of the patient as well as liver function parameters did not have any impact on the pharmacokinetic parameters to a statistically significant extent in the study population.Only CYP2C19 phenotype was considered to have a significant influence on V_max_.The final model with this covariate decreased the OFV, AIC and BIC by 17.55, 13.55 and 5.44 units, respectively.When the base model was updated with the final model, the number of parameters was increased from 11 to 13. The detailed PopPK parameter estimates derived from the final model are given in Table 2.

**TABLE 2:** Pharmacokinetic parameter estimates from the final joint model and bootstrap results.

Parameter	Final model		Bootstrap		Bias %
	Estimate	SE (%)	Median	95% CI	
F	0.895	Fix	—	—	—
K_a_ (h^−1^)	1.1	Fix	—	—	—
V_1_ (L)	207.29	33.76	213.82	71.52–356.12	3.15
CL_1_ (L/h)	1.91	26.68	1.99	0.67–3.33	4.19
V_2_(L)	10.01	28.78	9.37	3.18–14.82	−6.39
V_max_ (mg/h)	18.80	17.00	17.65	11.73–23.45	−6.12
K_m_	1.15	Fix	—	—	—
CL_2_ (L/h)	4.65	16.45	4.46	3.73–5.15	−4.09
I_max_	0.75	Fix	—	—	—
IC_50_(mg/L)	14.6	Fix	—	—	—
θ_NM_	0	Fix	—	—	—
θ_IM_	−0.31	42.10	−0.30	−0.47 to −0.13	3.23
θ_PM_	−0.61	28.37	−0.63	−1.19 to −0.06	−3.28
Interindividual variability					
ω_V1_ (%)	240.77	27.57	264.06	119.14–408.98	9.67
ω_CL1_ (%)	6.02	21.10	5.97	3.38–8.56	−0.83
ω_CL2_ (%)	25.57	24.25	24.15	12.31–35.99	−5.55
ω_Vmax_ (%)	21.13	14.78	20.69	10.85–30.53	−2.08
Residual variability					
VCZ-σ(%)	46.97	9.40	46.92	38.20–54.19	−0.11
VNO-σ(%)	27.93	6.16	27.91	22.95–33.49	−0.07

SE, standard error; F, oral bioavailability; Ka, absorption rate constant; V1, volume of distribution in the central compartment; CL1, the clearance of VCZ, other than the metabolic pathway converting to VNO; V2, volume of distribution in the metabolite compartment; Vmax, maximum elimination rate; Km, Michaelis-Menten constant; CL2, the clearance of VNO; ωV1, ωCL1, ωCL2, ωVmax: square root of interindividual variance for pharmacokinetic parameters; σ, residual variability.

a $V_{\max}=18.80\times \exp\left(\theta_{CYP2C19}\right)$ , θ_CYP2C19_ is equal to θ_NM_, θIM, or $C_{2}=\frac{A_{2}}{V_{2}}$ .

b Bias = (median estimate from bootstrap analysis–estimate from the final model)/estimate from the final model.

**FIGURE 3:**
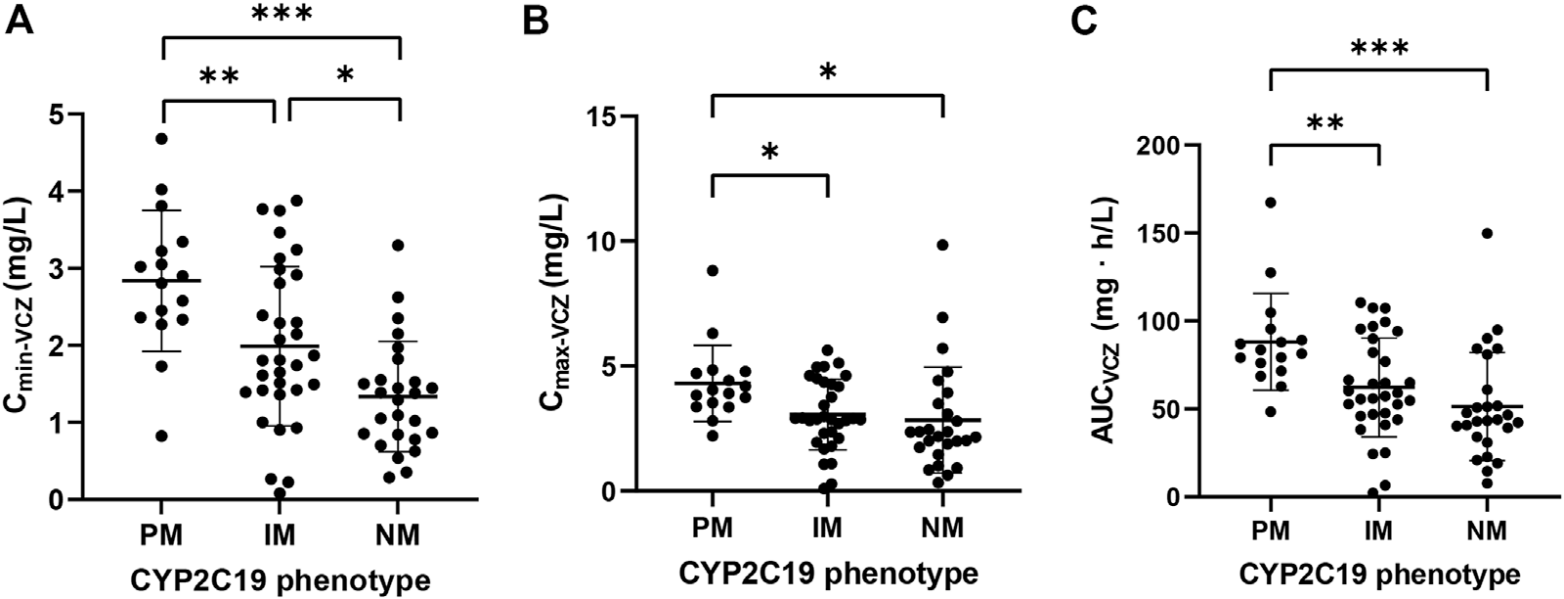
Comparison of **(A)** C_min-VCZ_, **(B)** C_max-VCZ_ and **(C)** AUC_VCZ_ in patients with different CYP2C19 phenotypes. Comparison of pharmacokinetic parameters in patients with different CYP2C19 phenotypes. The data were expressed as mean with standard error of the mean (*p < 0.05, **p < 0.01, ***p < 0.001).

The exposure parameters of VCZ and VNO estimated from the final joint model are presented in Supplementary Table S1. The one-way ANOVA and LSD test were used to compare the pharmacokinetic parameters in three groups classified according to CYP2C19 phenotypes. As shown in Figure 3, the result revealed remarkable differences in VCZ exposure (C_min-VCZ_, C_max-VCZ_, and AUC_VCZ_) among the three groups (*p* < 0.05).

### Model Validation

**FIGURE 4:**
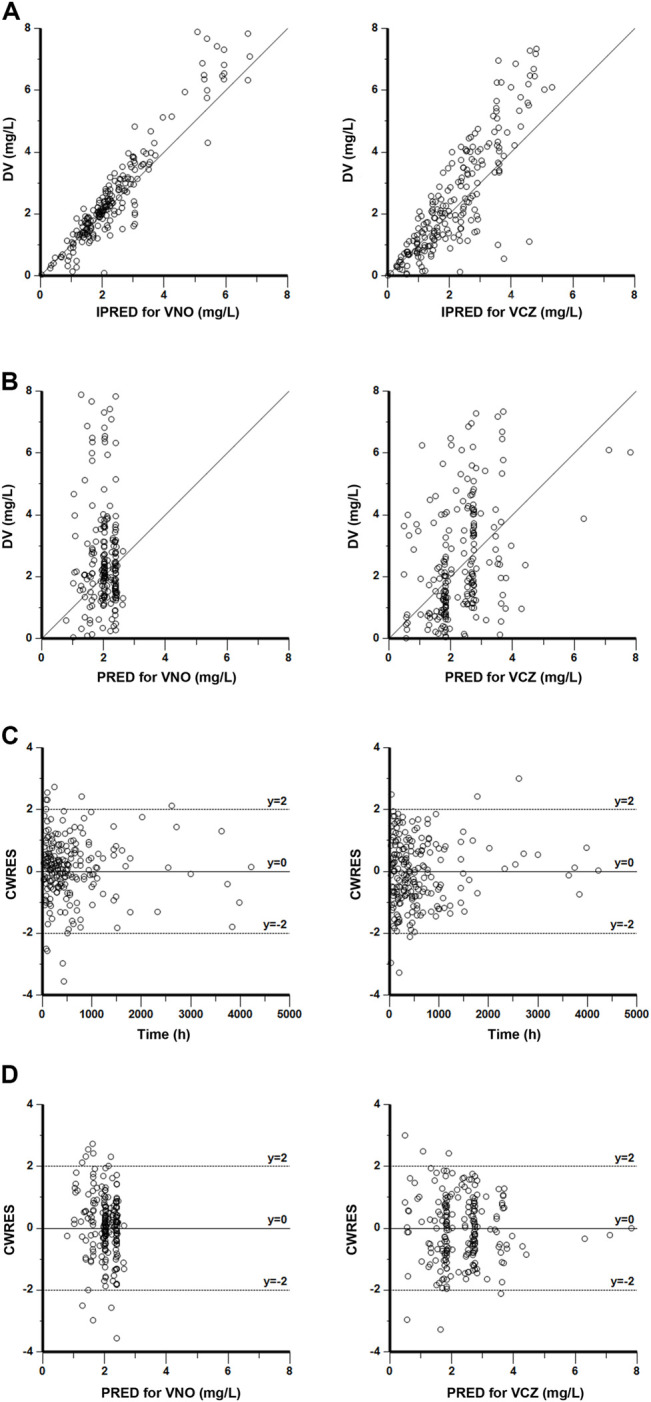
Goodness-of-fit plots of the final model for VNO (left panel) and VCZ (right panel) **(A)** Observations (DV) versus individual population predictions (IPRED) **(B)** DV versus population predictions (PRED) **(C)** Conditional weighted residuals (CWRES) versus time **(D)** CWRES versus PRED.

**FIGURE 5:**
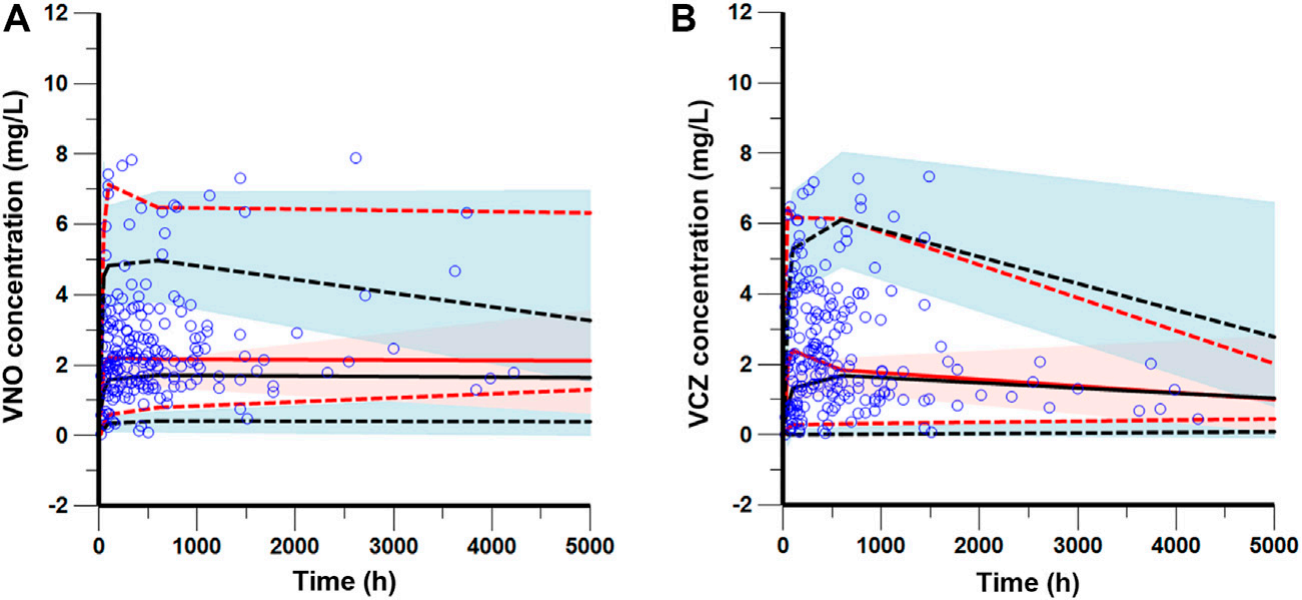
Visual predictive check of the final model for VNO **(A)** and VCZ **(B)**. The blue circles represent the observed data. The solid and dashed red lines represent the median, 2.5th percentile, and 97.5th percentile of the observed data, respectively. The solid and dashed black lines represent the median, 2.5th percentile, and 97.5th percentile of the simulated data, respectively. The shaded areas show the 95% predicted intervals of the 2.5th, 50th and 97.5th percentiles of the simulated data, respectively.

The goodness-of-fit plots presented in Figure 4 revealed acceptable agreement between the predicted and observed concentrations. The CWRES were generally distributed around zero with no trend, and the majority of them were in the -3 to +3 range. The results of bootstrap analysis are summarized in Table 2. All parameter estimates obtained from the final PopPK model laid within the 95% confidence intervals (CIs) calculated using the bootstrap method and were close to the median values with small bias (<10%), suggesting the robust stability of the final model. The VPC plots depicted in Figure 5 showed that the model performed well in predicting the plasma concentrations of both VCZ and VNO, and no evidence of model misspecification was found.

### Dosing Simulations

**FIGURE 6:**
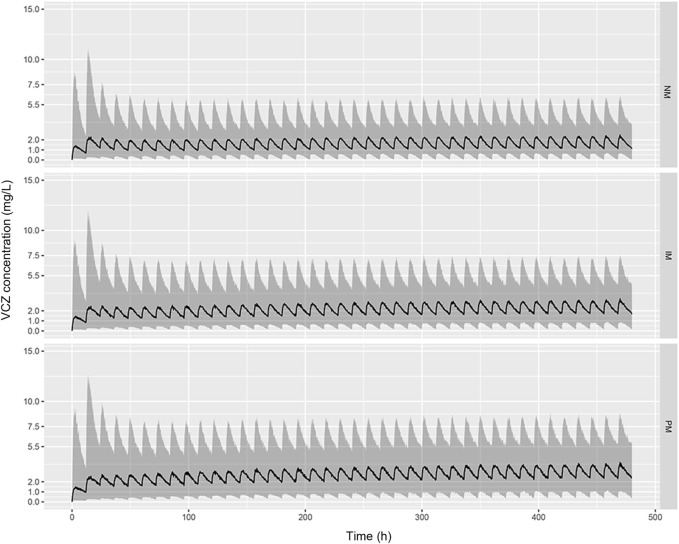
Predicted pharmacokinetic profiles of VCZ during the first 20 days of treatment obtained from simulated patients with CYP2C19 NMs **(A)**, CYP2C19 IMs **(B)**, and CYP2C19 PMs **(C)**. Recommended maintenance dosing regimen (200 mg, twice daily) was used for all patients. The black solid line represents the median of the simulated data, and the grey shaded area represents the prediction interval (10–90% confidence interval).

The predicted VCZ concentration-time profiles during 20 days after administration of standard doses (400 mg BID for two doses followed by 200 mg BID) were simulated according to the CYP2C19 phenotypes. As shown in Figure 6, the trough concentrations of VCZ at steady state attained the therapeutic range of 2–5.5 mg/L in PMs, whereas the trough levels in the subjects with IM and NM genotypes were below the therapeutic range. The assessment of the probability of achieving target through level of >2 mg/L with 200 mg twice daily oral dosing regimen indicated that up to 70.31% of NMs, 54.48% of IMs and 41.71% of PMs failed to reach the lower end of the therapeutic range. These results suggested that dosage adjustment based on CYP2C19 phenotype was needed to attain adequate exposure and to improve clinical response.

Table 3 shows the probability of VCZ target C_min_ attainment for different dosage regimens stratified by CYP2C19 phenotype. Considering the trade-offs between toxicity and efficacy, we concluded that the following regimens were appropriate for patients with different CYP2C19 phenotypes: 325 mg bid or 200 mg tid for NM patients, 275 mg bid or 175 mg tid for IM patients, and 225 mg bid or 150 mg tid for PM patients. The results also demonstrated that a lower C_min_ target of >1 mg/L would lead to higher PTA.

**TABLE 3:** Probability of VCZ target trough concentration attainment from model simulations.

CYP2C19 phenotypes	Maintenance dose of VCZ	Median of C_ssmin_ (mg/L)	PTA (%)		
			C_ssmin_≥ 1 mg/L	C_ssmin_≥ 2 mg/L	C_ssmin_≥ 5.5 mg/L
NM	200 mg, bid	1.20	56.09	29.69	2.48
	300 mg, bid	2.73	78.36	61.06	19.78
	325 mg, bid	3.20	81.05	66.11	25.76
	350 mg, bid	3.70	83.59	70.76	32.00
	400 mg, bid	4.79	86.91	77.73	43.66
	200 mg, tid	3.01	82.27	65.52	22.59
	250 mg, tid	4.62	88.69	78.50	41.84
IM	200 mg, bid	1.79	69.08	45.52	6.12
	250 mg, bid	2.70	79.58	61.36	17.50
	275 mg, bid	3.21	82.74	67.43	23.95
	300 mg, bid	3.74	85.21	72.47	30.81
	400 mg, bid	6.07	90.36	83.82	54.67
	175 mg, tid	3.21	84.95	68.83	23.32
	200 mg, tid	4.06	88.35	76.32	34.34
PM	200 mg, bid	2.44	78.83	58.29	11.99
	225 mg, bid	2.97	82.81	65.94	19.30
	250 mg, bid	3.53	85.56	71.70	26.56
	300 mg, bid	4.68	89.14	79.42	41.36
	125 mg, tid	2.39	80.20	58.08	10.56
	150 mg, tid	3.21	86.04	70.06	21.63
	175 mg, tid	4.09	89.56	77.86	34.00

VCZ, voriconazole; C_ssmin_, VCZ, trough concentration atsteadystate; PTA, the probability of target attainment; NM, normal metabolizer; IM, intermediate metabolizer; PM, poor metabolizer.

## Discussion

In most studies conducted previously, only the pharmacokinetics of VCZ had been described. However, pharmacokinetic studies focused on its main metabolites were relatively few. To the best of our knowledge, this is the first report to simultaneously evaluate the PopPK characteristics of VCZ and its N-oxide metabolite using a mechanistic model. In the current study, VCZ disposition was well described by a one-compartment model with first-order absorption and mixed linear and concentration-dependent nonlinear elimination. While VNO pharmacokinetics was characterized by a one-compartment model with first-order elimination as an extension of the parent drug (VCZ) model. Most studies reported that the one- or two-compartment model with combined first-order absorption and linear elimination well fitted VCZ data in adults (Lin et al., 2018; Chen et al., 2019; Wang et al., 2021). Other studies indicated that VCZ displayed nonlinear pharmacokinetics with saturable clearance (Dolton et al., 2014; Mangal et al., 2018). Meanwhile, Liu et al. and Friberg et al. suggested two-compartment models with mixed linear and time-dependent nonlinear elimination for VCZ disposition (Friberg et al., 2012; Liu and Mould, 2014). We have tried to add a peripheral compartment to the structural model, but this resulted in large prediction errors for pharmacokinetic parameters. Therefore, a one-compartment model seems best suited for VCZ pharmacokinetics in the present study. We also tried to describe the auto-inhibitory characteristics of VCZ or VNO using the time or concentration-dependent model. However, it has been reported that no significant auto-inhibitory effect was observed after the first day of administration (Friberg et al., 2012). Considering the fact that only three samples were collected in the present study during the first 24 h of administration, the time-dependent model may not be suitable to reflect the auto-inhibition characteristics of VCZ. Then, the auto-inhibition effect of VNO was incorporated into the structural model in a concentration-dependent manner. As the observed VNO pharmacokinetic data in our study do not cover the entire concentration range of the contention-auto-inhibition curve, the parameters related with auto-inhibition cannot be properly estimated based on the observed pharmacokinetic data in this study. To resolve this limitation, we fixed k_m_, I_max_, and IC_50_ based on the modeling results from previous literature (Liu et al., 2014; Hohmann et al., 2017).

Body weight, liver function and concomitant medications were commonly identified variables in previous PopPK analyses (Shi et al., 2019). These potential covariates were investigated in our model, but no statistically significant impact was found on the VCZ pharmacokinetic profiles. The study carried out by Liu et al. showed only a slight association between WT and VCZ exposure in adults (Liu and Mould, 2014). This might account for the failure to incorporate WT into the final model in our study. As reported in the literature, WT has little or no effect on VCZ elimination, which did not support the use of weight-based dosing strategies in adult patients (Han et al., 2010). Moreover, numerous pharmacokinetic studies have indicated that liver function had a significant impact on VCZ elimination (Wang et al., 2014; Chen et al., 2015; Li et al., 2017). However, in the current study, liver function test results demonstrated that most patients had mild liver dysfunction according to the National Cancer Institute Organ Dysfunction Working Group (NCI-ODWG) criteria. Hence, the effect of liver function on VCZ clearance was limited in the study and might be masked by other more significant variables. Theoretically, co-medication with proton pump inhibitors (PPIs) or glucocorticoids might affect the metabolic activity of CYP2C19 and further altered VCZ exposure. Jia et al. revealed that glucocorticoids reduced the C _min_/dose levels of VCZ (Jia et al., 2021). But to date, the effect of above-mentioned drugs on the disposition of VCZ remains controversial. It is worth noting that sample size, administered dose, and the type of PPI or glucocorticoid may be influential factors on VCZ exposure (Cojutti et al., 2016). In the present study, neither PPIs nor glucocorticoids seemed to affect the pharmacokinetic parameters of VCZ. The reason may be that patients were treated with relatively low daily doses of omeprazole or different glucocorticoids.

Among various covariates influencing the disposition of VCZ, CYP2C19 genotype was the most important factor contributing to the high variability of pharmacokinetics, and was identified as a determinant variable for nonlinear clearance and exposure of VCZ. In the current study, the V_max_ of VCZ decreased by 26.7 and 45.7% in patients with CYP2C19 IM and PM genotypes, respectively, when compared to patients carrying CYP2C19 NM genotypes. This result was generally in agreement with a previous study showing a 41.2% reduction of V_max_ in patients with CYP2C19 loss-of-function alleles (Dolton et al., 2014). On the other hand, the exposure parameters of VCZ (C_min_, C_max_, AUC) tended to be increased in the following order: CYP2C19NMs < IMs < PMs. The C_min_ of VCZ in CYP2C19 PMs was approximately 40% and 110% higher than that in CYP2C19 IMs and NMs, respectively. Additionally, there was no significant difference in the VNO exposure among the three CYP2C19 phenotypes, which could be due to the involvement of other metabolizing enzymes in the N-oxide metabolic process (Murayama et al., 2007).

For patients carrying different CYP2C19 genotypes, the MR of VNO to VCZ was 1.11 ± 0.94. In contrast to VCZ exposure, MR was found to be increased in the order of CYP2C19PMs < IMs < NMs. However, no statistical difference was observed in MR between the three groups. This may be due to the limited sample size in our study. The VNO exposures were similar across different CYP2C19 metabolic phenotypes, so it could be speculated that the higher MR in NM patients than in PM patients was not attributed to more VCZ being converted to its N-oxide metabolite, but probably due to the decreased VCZ exposure in NMs compared to PMs. Although plasma level monitoring of VNO is not routinely recommended, determination of MR may play a role in evaluation of metabolic capacity of VCZ and provide information about the CYP2C19 metabolic phenotype in patients (Gómez-López, 2020).

The standard oral dosing regimen of VCZ is suggested as two loading doses of 400 mg q12 h followed by a maintenance dose of 200 mg twice daily for adult patients regardless of CYP2C19 genotypes. The CPIC guideline recommends the use of alternative antifungal agents that is not dependent on CYP2C19 metabolism for poor, rapid, and ultra-rapid metabolizers. For IMs, the dose-adjusted C_mins_ of VCZ may be higher than that in NMs. The simulation results demonstrated that recommended maintenance dosing of 200 mg twice daily was not sufficient for the patients carrying different CYP2C19 genotypes to achieve the VCZ therapeutic range (2–5.5 mg/L). Dosing regimens suggested in our study could result in a relatively higher proportion of NM, IM and PM patients with adequate VCZ concentration during treatment. However, the probabilities of exceeding potentially toxic concentration were predicted to be up to around 23% in these patients. In different clinical scenarios, the likelihood of successful treatment and the risk of drug-related toxicities should be considered comprehensively, and then dosage modification or alternative medication could be selected according to the patient’s clinical situation.

Several limitations in our study should be noted. First, only trough concentrations were obtained, which made it difficult to estimate pharmacokinetic parameters in absorption and distribution phase. Additionally, the MR of VNO to VCZ could only partially explain the metabolic capacity and nonlinear pharmacokinetics of VCZ in the present study, so the evaluation for other metabolites of VCZ (e.g., hydroxides) is warranted in further study.

## Conclusion

In conclusion, a joint PopPK model developed here for the pharmacokinetics of VCZ and VNO well described the concentration profiles in immunocompromised patients. The covariate screening results demonstrated that VCZ pharmacokinetics were significantly influenced by the CYP2C19 genotype, which was identified to be a major determinant for VCZ exposure. The proposed CYP2C19 phenotype-guided maintenance dosing regimens could provide a theoretical basis for dosage individualization to improve clinical outcomes and minimize drug-related toxicities, while confirmation through clinical studies is still necessary.

## Data Availability

The original contributions presented in the study are included in the article/Supplementary Material, further inquiries can be directed to the corresponding authors.

## References

[B1] Ashbee H. R. Barnes R. A. Johnson E. M. Richardson M. D. Gorton R. Hope W. W. (2014). Therapeutic Drug Monitoring (TDM) of Antifungal Agents: Guidelines from the British Society for Medical Mycology. J. Antimicrob. Chemother. 69 (5), 1162–1176. 10.1093/jac/dkt508 24379304 PMC3977608

[B2] Chawla P. K. Nanday S. R. Dherai A. J. Soman R. Lokhande R. V. Naik P. R. (2015). Correlation of CYP2C19 Genotype with Plasma Voriconazole Levels: a Preliminary Retrospective Study in Indians. Int. J. Clin. Pharm. 37 (5), 925–930. 10.1007/s11096-015-0143-y 26024717

[B3] Chen C. Yang T. Li X. Ma L. Liu Y. Zhou Y. (2019). Population Pharmacokinetics of Voriconazole in Chinese Patients with Hematopoietic Stem Cell Transplantation. Eur. J. Drug Metab. Pharmacokinet. 44 (5), 659–668. 10.1007/s13318-019-00556-w 31041728

[B4] Chen W. Xie H. Liang F. Meng D. Rui J. Yin X. (2015). Population Pharmacokinetics in China: The Dynamics of Intravenous Voriconazole in Critically Ill Patients with Pulmonary Disease. Biol. Pharm. Bull. 38 (7), 996–1004. 10.1248/bpb.b14-00768 26133710

[B5] Cojutti P. Candoni A. Forghieri F. Isola M. Zannier M. E. Bigliardi S. (2016). Variability of Voriconazole Trough Levels in Haematological Patients: Influence of Comedications with Cytochrome P450(CYP) Inhibitors And/or with CYP Inhibitors Plus CYP Inducers. Basic Clin. Pharmacol. Toxicol. 118 (6), 474–479. 10.1111/bcpt.12530 26572687

[B6] Dean L. (2012). “Voriconazole Therapy and CYP2C19 Genotype,” in Medical Genetics Summaries. Editors Pratt V. M. Scott S. A. Pirmohamed M. Esquivel B. Kane M. S. Kattman B. L. (Bethesda (MD)): National Center for Biotechnology Information (US).

[B7] Dolton M. J. Mikus G. Weiss J. Ray J. E. McLachlan A. J. (2014). Understanding Variability with Voriconazole Using a Population Pharmacokinetic Approach: Implications for Optimal Dosing. J. Antimicrob. Chemother. 69 (6), 1633–1641. 10.1093/jac/dku031 24554646

[B8] Dolton M. J. Ray J. E. Chen S. C. Ng K. Pont L. G. McLachlan A. J. (2012). Multicenter Study of Voriconazole Pharmacokinetics and Therapeutic Drug Monitoring. Antimicrob. Agents Chemother. 56 (9), 4793–4799. 10.1128/aac.00626-12 22751544 PMC3421881

[B9] Fisher B. T. Robinson P. D. Lehrnbecher T. Steinbach W. J. Zaoutis T. E. Phillips B. (2018). Risk Factors for Invasive Fungal Disease in Pediatric Cancer and Hematopoietic Stem Cell Transplantation: A Systematic Review. J. Pediatr. Infect Dis Soc 7 (3), 191–198. 10.1093/jpids/pix030 PMC12427443 28549148

[B10] Friberg L. E. Ravva P. Karlsson M. O. Liu P. (2012). Integrated Population Pharmacokinetic Analysis of Voriconazole in Children, Adolescents, and Adults. Antimicrob. Agents Chemother. 56 (6), 3032–3042. 10.1128/aac.05761-11 22430956 PMC3370730

[B11] Fu L. Q. Huang F. Wu D. Z. Guo J. H. (2004). Comparison of Genetic Polymorphism of Cytochrome CYP2C19 between Men and Women in Chinese Population. Yao Xue Xue Bao 39 (3), 161–163. 15171646

[B12] Ghannoum M. A. Kuhn D. M. (2002). Voriconazole -- Better Chances for Patients with Invasive Mycoses. Eur. J. Med. Res. 7 (5), 242–256. 12069915

[B13] Gómez-López A. (2020). Antifungal Therapeutic Drug Monitoring: Focus on Drugs without a clear Recommendation. Clin. Microbiol. Infect. 26 (11), 1481–1487. 10.1016/j.cmi.2020.05.037 32535150

[B14] Han K. Capitano B. Bies R. Potoski B. A. Husain S. Gilbert S. (2010). Bioavailability and Population Pharmacokinetics of Voriconazole in Lung Transplant Recipients. Antimicrob. Agents Chemother. 54 (10), 4424–4431. 10.1128/aac.00504-10 20679503 PMC2944566

[B15] He L. Chen S. Li J. Xie X. Huang L. Kuang Y. (2020). Genetic and Phenotypic Frequency Distribution of CYP2C9, CYP2C19 and CYP2D6 in over 3200 Han Chinese. Clin. Exp. Pharmacol. Physiol. 47 (10), 1659–1663. 10.1111/1440-1681.13357 32469422

[B16] Hohmann N. Kreuter R. Blank A. Weiss J. Burhenne J. Haefeli W. E. (2017). Autoinhibitory Properties of the Parent but Not of the N-Oxide Metabolite Contribute to Infusion Rate-dependent Voriconazole Pharmacokinetics. Br. J. Clin. Pharmacol. 83 (9), 1954–1965. 10.1111/bcp.13297 28370390 PMC5555860

[B17] Jeu L. Piacenti F. J. Lyakhovetskiy A. G. Fung H. B. (2003). Voriconazole. Clin. Ther. 25 (5), 1321–1381. 10.1016/s0149-2918(03)80126-1 12867215

[B18] Jia S. J. Gao K. Q. Huang P. H. Guo R. Zuo X. C. Xia Q. (2021). Interactive Effects of Glucocorticoids and Cytochrome P450 Polymorphisms on the Plasma Trough Concentrations of Voriconazole. Front. Pharmacol. 12, 666296. 10.3389/fphar.2021.666296 34113252 PMC8185288

[B19] Jin H. Wang T. Falcione B. A. Olsen K. M. Chen K. Tang H. (2016). Trough Concentration of Voriconazole and its Relationship with Efficacy and Safety: a Systematic Review and Meta-Analysis. J. Antimicrob. Chemother. 71 (7), 1772–1785. 10.1093/jac/dkw045 26968880 PMC4896404

[B20] Lehrnbecher T. Fisher B. T. Phillips B. Beauchemin M. Carlesse F. Castagnola E. (2020). Clinical Practice Guideline for Systemic Antifungal Prophylaxis in Pediatric Patients with Cancer and Hematopoietic Stem-Cell Transplantation Recipients. J. Clin. Oncol. 38 (27), 3205–3216. 10.1200/jco.20.00158 32459599 PMC7499615

[B21] Li Z. W. Peng F. H. Yan M. Liang W. Liu X. L. Wu Y. Q. (2017). Impact of CYP2C19 Genotype and Liver Function on Voriconazole Pharmacokinetics in Renal Transplant Recipients. Ther. Drug Monit. 39 (4), 422–428. 10.1097/ftd.0000000000000425 28604474 PMC5538305

[B22] Lin X. B. Li Z. W. Yan M. Zhang B. K. Liang W. Wang F. (2018). Population Pharmacokinetics of Voriconazole and CYP2C19 Polymorphisms for Optimizing Dosing Regimens in Renal Transplant Recipients. Br. J. Clin. Pharmacol. 84 (7), 1587–1597. 10.1111/bcp.13595 29607533 PMC6005582

[B23] Liu P. Mould D. R. (2014). Population Pharmacokinetic-Pharmacodynamic Analysis of Voriconazole and Anidulafungin in Adult Patients with Invasive Aspergillosis. Antimicrob. Agents Chemother. 58 (8), 4727–4736. 10.1128/aac.02809-13 24914120 PMC4126689

[B24] Mafuru M. Wu S. He S. Lu X. Huang J. Jiang H. (2019). The Influence of Proinflammatory Cytokines on Voriconazole Trough Concentration in Patients with Different Forms of Hematologic Disorders. J. Clin. Pharmacol. 59 (10), 1340–1350. 10.1002/jcph.1422 30997931

[B25] Mangal N. Hamadeh I. S. Arwood M. J. Cavallari L. H. Samant T. S. Klinker K. P. (2018). Optimization of Voriconazole Therapy for the Treatment of Invasive Fungal Infections in Adults. Clin. Pharmacol. Ther. 104 (5), 957–965. 10.1002/cpt.1012 29315506 PMC6037619

[B26] Moriyama B. Obeng A. O. Barbarino J. Penzak S. R. Henning S. A. Scott S. A. (2017). Clinical Pharmacogenetics Implementation Consortium (CPIC) Guidelines for CYP2C19 and Voriconazole Therapy. Clin. Pharmacol. Ther. 102 (1), 45–51. 10.1002/cpt.583 27981572 PMC5474211

[B27] Murayama N. Imai N. Nakane T. Shimizu M. Yamazaki H. (2007). Roles of CYP3A4 and CYP2C19 in Methyl Hydroxylated and N-Oxidized Metabolite Formation from Voriconazole, a New Anti-fungal Agent, in Human Liver Microsomes. Biochem. Pharmacol. 73 (12), 2020–2026. 10.1016/j.bcp.2007.03.012 17433262

[B28] Pascual A. Csajka C. Buclin T. Bolay S. Bille J. Calandra T. (2012). Challenging Recommended Oral and Intravenous Voriconazole Doses for Improved Efficacy and Safety: Population Pharmacokinetics-Based Analysis of Adult Patients with Invasive Fungal Infections. Clin. Infect. Dis. 55 (3), 381–390. 10.1093/cid/cis437 22610925

[B29] Schulz J. Kluwe F. Mikus G. Michelet R. Kloft C. (2019). Novel Insights into the Complex Pharmacokinetics of Voriconazole: a Review of its Metabolism. Drug Metab. Rev. 51 (3), 247–265. 10.1080/03602532.2019.1632888 31215810

[B30] Shi C. Xiao Y. Mao Y. Wu J. Lin N. (2019). Voriconazole: A Review of Population Pharmacokinetic Analyses. Clin. Pharmacokinet. 58 (6), 687–703. 10.1007/s40262-019-00735-7 30687893

[B31] Sim S. C. Risinger C. Dahl M. L. Aklillu E. Christensen M. Bertilsson L. (2006). A Common Novel CYP2C19 Gene Variant Causes Ultrarapid Drug Metabolism Relevant for the Drug Response to Proton Pump Inhibitors and Antidepressants. Clin. Pharmacol. Ther. 79 (1), 103–113. 10.1016/j.clpt.2005.10.002 16413245

[B32] Stott K. E. Hope W. W. (2017). Therapeutic Drug Monitoring for Invasive Mould Infections and Disease: Pharmacokinetic and Pharmacodynamic Considerations. J. Antimicrob. Chemother. 72 (Suppl. l_1), i12–i18. 10.1093/jac/dkx029 28355463

[B33] Sung L. Gamis A. Alonzo T. A. Buxton A. Britton K. Deswarte-Wallace J. (2009). Infections and Association with Different Intensity of Chemotherapy in Children with Acute Myeloid Leukemia. Cancer 115 (5), 1100–1108. 10.1002/cncr.24107 19156894 PMC2677372

[B34] Theuretzbacher U. Ihle F. Derendorf H. (2006). Pharmacokinetic/pharmacodynamic Profile of Voriconazole. Clin. Pharmacokinet. 45 (7), 649–663. 10.2165/00003088-200645070-00002 16802848

[B35] Vena A. Muñoz P. Mateos M. Guinea J. Galar A. Pea F. (2020). Therapeutic Drug Monitoring of Antifungal Drugs: Another Tool to Improve Patient Outcome? Infect. Dis. Ther. 9 (1), 137–149. 10.1007/s40121-020-00280-y 32026399 PMC7054538

[B36] Wang J. H. Li P. Q. Fu Q. Y. Li Q. X. Cai W. W. (2007). Cyp2c19 Genotype and Omeprazole Hydroxylation Phenotype in Chinese Li Population. Clin. Exp. Pharmacol. Physiol. 34 (5-6), 421–424. 10.1111/j.1440-1681.2007.04583.x 17439410

[B37] Wang T. Chen S. Sun J. Cai J. Cheng X. Dong H. (2014). Identification of Factors Influencing the Pharmacokinetics of Voriconazole and the Optimization of Dosage Regimens Based on Monte Carlo Simulation in Patients with Invasive Fungal Infections. J. Antimicrob. Chemother. 69 (2), 463–470. 10.1093/jac/dkt369 24084636

[B38] Wang T. Yan M. Tang D. Dong Y. Zhu L. Du Q. (2021). Using Child-Pugh Class to Optimize Voriconazole Dosage Regimens and Improve Safety in Patients with Liver Cirrhosis: Insights from a Population Pharmacokinetic Model-Based Analysis. Pharmacotherapy 41 (2), 172–183. 10.1002/phar.2474 33064889

[B39] Weiss J. Ten Hoevel M. M. Burhenne J. Walter-Sack I. Hoffmann M. M. Rengelshausen J. (2009). CYP2C19 Genotype Is a Major Factor Contributing to the Highly Variable Pharmacokinetics of Voriconazole. J. Clin. Pharmacol. 49 (2), 196–204. 10.1177/0091270008327537 19033450

[B40] Yamada T. Mino Y. Yagi T. Naito T. Kawakami J. (2015). Saturated Metabolism of Voriconazole N-Oxidation Resulting in Nonlinearity of Pharmacokinetics of Voriconazole at Clinical Doses. Biol. Pharm. Bull. 38 (10), 1496–1503. 10.1248/bpb.b15-00241 26424015

[B41] Yi W. M. Schoeppler K. E. Jaeger J. Mueller S. W. MacLaren R. Fish D. N. (2017). Voriconazole and Posaconazole Therapeutic Drug Monitoring: a Retrospective Study. Ann. Clin. Microbiol. Antimicrob. 16 (1), 60. 10.1186/s12941-017-0235-8 28893246 PMC5594434

[B42] Zuo J. Xia D. Jia L. Guo T. (2012). Genetic Polymorphisms of Drug-Metabolizing Phase I Enzymes CYP3A4, CYP2C9, CYP2C19 and CYP2D6 in Han, Uighur, Hui and Mongolian Chinese Populations. Pharmazie 67 (7), 639–644. 22888523

