# Supplementary material for: Application of Population Pharmacokinetic Analysis to Characterize CYP2C19 Mediated Metabolic Mechanism of Voriconazole and Support Dose Optimization
Source: Front Pharmacol. 2022 Jan 3;12:730826. doi: 10.3389/fphar.2021.730826 (PMC8762230; doi:10.3389/fphar.2021.730826)
Supplement: Supplementary file 1 [file Table1.docx]

Supplementary Material

Table S1 Voriconazole and voriconazole-N-oxide pharmacokinetic parameters in patients with different CYP2C19 phenotypes.

| Parameter | NM (n=26) | IM (n=32) | PM (n=16) | *p value* | | |
| --- | --- | --- | --- | --- | --- | --- |
|  |  |  |  | PM vs. IM | PM vs. EM | IM vs. EM |
| C_min-VCZ_ (μg/ml) | 1.34 (0.72) | 1.99 (1.03) | 2.84 (0.91) | 0.003 | < 0.001 | 0.008 |
| C_max-VCZ_ (μg/ml) | 2.83 (2.12) | 3.06 (1.41) | 4.30 (1.52) | 0.020 | 0.009 | 0.627 |
| C_min-VNO_ (μg/ml) | 2.22 (1.43) | 2.01 (0.94) | 2.51 (1.51) | 0.203 | 0.478 | 0.531 |
| C_max-VNO_ (μg/ml) | 2.49 (1.45) | 2.19 (0.95) | 2.64 (1.53) | 0.263 | 0.727 | 0.378 |
| AUC_VCZ_ (mg·h/l) | 51.37 (30.77) | 62.18 (28.11) | 88.10 (27.54) | 0.005 | < 0.001 | 0.162 |
| AUC_VNO_ (mg·h/l) | 57.46 (34.56) | 51.09 (22.58) | 62.14 (36.56) | 0.239 | 0.629 | 0.431 |
| MR | 1.30 (0.64) | 1.14 (1.24) | 0.74 (0.48) | 0.169 | 0.062 | 0.507 |

Note: Data are reported as mean±SD.

NM, normal metabolizer; IM, intermediate metabolizer; PM, poor metabolizer; Cmin-VCZ, VCZ trough concentration; Cmax-VCZ, VCZ peak concentration; Cmin-VNO, VNO trough concentration; Cmax-VNO, VNO peak concentration; AUCVCZ, the area under VCZ plasma concentration-time curve; AUCVNO, the area under VNO plasma concentration-time curve, MR, metabolic ratio.
